# Psychometric evaluation of the Swedish version of the PROMIS Sexual Function and Satisfaction Measures in clinical and nonclinical young adult populations

**DOI:** 10.1093/sexmed/qfac006

**Published:** 2023-01-12

**Authors:** Emma Hovén, Kathryn E Flynn, Kevin P Weinfurt, Lars E Eriksson, Lena Wettergren

**Affiliations:** Department of Women’s and Children’s Health, Uppsala University, Uppsala, Sweden; Department of Women’s and Children’s Health, Karolinska Institutet, Stockholm, Sweden; Department of Medicine, Medical College of Wisconsin, Milwaukee, WI, United States; Department of Population Health Sciences, Duke University School of Medicine, Durham, NC, United States; Department of Neurobiology, Care Sciences and Society, Karolinska Institutet, Huddinge, Sweden; School of Health and Psychological Sciences, University of London, London, United Kingdom; Medical Unit Infectious Diseases, Karolinska University Hospital, Huddinge, Sweden; Department of Women’s and Children’s Health, Karolinska Institutet, Stockholm, Sweden; Department of Public Health and Caring Sciences, Uppsala University, Uppsala, Sweden

**Keywords:** Sexual satisfaction, Sexual health, Sexual dysfunction, Erectile dysfunction, Psychometrics, Patient-reported outcome measures

## Abstract

**Background:**

The Patient-Reported Outcomes Measurement Information System (PROMIS®) Sexual Function and Satisfaction (SexFS) version 2.0 measurement tool was developed to assess sexual functioning and satisfaction in the general population regardless of health condition and sexual orientation.

**Aim:**

The study aimed to evaluate the psychometric properties of the Swedish version of the PROMIS SexFS measure in clinical and nonclinical populations of young adults (aged <40 years).

**Methods:**

The SexFS was answered by a clinical population of young adult women (*n* = 180) and men (*n* = 110) with breast cancer and testicular cancer, respectively, and a nonclinical population of young adult women (*n* = 511) and men (*n* = 324) from the general population. Psychometric properties were evaluated by examining data quality (score distribution, floor and ceiling effects, proportion of missing data), construct validity (corrected item, total correlation, scaling success), and reliability (Cronbach α).

**Outcomes:**

The following domains of the SexFS 2.0 were investigated: Vaginal Lubrication, Vaginal Discomfort, Vulvar Discomfort- Clitoral, Vulvar Discomfort- Labial, Erectile Function, Interest in Sexual Activity, Satisfaction With Sex Life, Orgasm– Ability, and Orgasm- Pleasure.

**Results:**

The Swedish version of the SexFS 2.0 generated data of acceptable quality. Some noteworthy floor or ceiling effects were identified across domains and respondent groups. Corrected item totals were used to express the coherence between an item and the other items in the domain. The correlation coefficients were above 0.40 for all items, except for 1 of the items within the Vaginal Discomfort domain and for the items in the Erectile Function domain in the nonclinical group of men. High proportions of scaling success were noted across domains (96%-100%). Reliability was satisfactory (α = 0.74-0.92) for all domains, expect for Erectile Function of the nonclinical group (α = 0.53), due to low variability in item responses, which was improved somewhat (α = 0.65) when combined with the clinical group.

**Clinical Implications:**

A flexible tool to measure self-reported sexual function and satisfaction in young men and women is available for researchers and clinicians in Sweden.

**Strengths and Limitations:**

The nationwide population-based sample of patients with cancer, identified from national quality registers, minimized selection bias. However, men in the general population had a lower response rate (34%) compared to the other groups, which introduced a risk of bias in estimates. The psychometric evaluation was limited to young adults (aged 19-40 years).

**Conclusion:**

The results provide evidence for the validity and reliability of the Swedish version of the SexFS measure for the assessment of sexual functioning and satisfaction in young adults from both clinical and nonclinical populations.

## Introduction

Sexual health, functioning and satisfaction are important for the overall health and well-being of individuals. Measurement methods to assess sexual function and satisfaction in a comprehensive, valid, and precise manner are necessary to evaluate and compare sexual outcomes and to develop interventions for those who experience or are at risk for impaired sexual functioning. Indeed, the need for high-quality patient-reported outcome measures (PROMs) of sexual function has been highlighted.^1–4^

The National Institutes of Health commenced the Patient-Reported Outcomes Measurement Information System (PROMIS®) with the goal to provide standardized and reliable PROMs. The National Cancer Institute identified sexual functioning as a key PROM domain for development, as this area can be affected by cancer and its treatments. Through the PROMIS network, this resulted in the first version of the Sexual Function and Satisfaction Measure (PROMIS SexFS), developed for assessment of sexual function and satisfaction in patients with cancer.^4^ Building on the thorough process of the development of SexFS, a revised version of the measure (SexFS v2.0) was developed that was based on a renewed and expanded literature review, input of clinical experts as well as focus groups, and interviews with patients.^5^ In addition to the need to expand and improve the concepts being measured, an important motivation for developing the SexFS v2.0 was for the measurement system to be suitable for the general population regardless of health condition and sexual orientation, and to provide meaningful normal values for the US adult population.^5^ The SexFS v2.0 has shown acceptable content validity, face validity, known-groups validity, and construct validity.^5^

In the preparatory phase of a larger research program investigating concerns regarding sexual function and fertility in young adults diagnosed with cancer in Sweden (Fex-Can),^6–8^ it became evident that at that time no comprehensive, valid, and reliable measure existed in Sweden for assessment of sexual function in adult women and men regardless of sexual orientation, including items for individuals not engaged in partner sex. The preliminary version of the PROMIS SexFS v2.0 was thus translated from English into Swedish according to the principles of the Functional Assessment of Chronic Illness Therapy (FACIT), FACIT translation (FACITrans), and PROMIS.^9^ However, to our knowledge, the psychometric properties of the Swedish version of the SexFS measure have not been previously reported. The overall objective of the present study was to evaluate the psychometric properties of the Swedish version of the SexFS measures in both clinical and nonclinical samples of men and women in Sweden.

## Methods

### The SexFS v2.0 measure

The SexFS v2.0 measurement system is used to assess multiple components of sexual functioning and satisfaction, covered by a range of domains and general screening items about, for example, sexual activity in the past 30 days.^5^ As opposed to the original version of the SexFS, items of the SexFS v2.0 are universal rather than cancer specific. Moreover, some domains are body-part specific (eg, Vaginal Discomfort and Erectile Function), while others are generic (eg, Interest in Sexual Activity). The measurement strategy was developed within an Item Response Theory (IRT) framework, though not all domains use IRT-based scoring.^10^ Reliable scores can be generated without having to administer all the items in a domain; therefore, researchers are encouraged to select items and domains that are relevant to their specific study population.

For all domains, higher scores indicate more of the thing described by the domain label. For example, higher scores on the Interest in Sexual Activity domain indicate more interest, higher scores on Erectile Function indicate better function, while higher scores on Vaginal Discomfort indicate more discomfort.

#### Translational and cultural adaption of the Swedish version of the SexFS v2.0

Selected items of the preliminary version of the PROMIS SexFS v2.0 domains (Supplementary Table 1) were translated from English into Swedish and culturally adapted in accordance with the FACIT translation methodology.^9^ The procedure consists of 2 independent forward translations followed by reconciliation and a blind back-translation of the reconciled version. The preliminary Swedish version was then reviewed by the language coordinator at the PROMIS Statistical Centre. At this step, ambiguous expressions were discussed to ensure equivalence of meaning and measurement with the original language before pretesting the new version in Sweden. Cognitive interviews were then conducted with a purposeful sample (*n* = 19) including men and women from the general population (*n* = 13) as well as patients with cancer (*n* = 6); sociodemographic characteristics are presented in Table 1. Overall, participants found the items, instructions, and response options comprehensive, and the interviews resulted in only minor modifications.^7^^,^^8^

**Table 1:** Characteristics of the participants in cognitive interviews to test the Swedish version of the PROMIS SexFS v2.0 measure.

	**Women**		**Men**	
	**Cancer population, *n* = 4**	**General population, *n* = 7**	**Cancer population, *n* = 2**	**General population, *n* = 6**
Age, years, *n*				
16-17	1	1	1	1
18-30	3	3	1	1
31-55	-	1	-	3
56-70	-	2	-	1
Country of birth, *n*				
Sweden	4	6	1	4
Other	-	1	-	2
Missing			1	
Education, *n*				
High school	1	2	1	1
University	2	4	-	3
Missing	1	1	1	2
Sexual orientation, *n*				
Heterosexual or straight	4	6	2	4
Gay, lesbian, or bisexual	-	1	-	2
Sexually active, *n*^a^				
Yes	2	5	1	6

a Sexually active, alone or with a partner.

The Swedish version evaluated in the present study consists of 15 possible items for women and 11 item for men, from the preliminary version 2 of the SexFS measure, including a general screener item and 1 to 4 items for each of the domains (Supplementary Table 1). The translated and tested domains and items were selected to adequately evaluate a psychoeducational intervention aiming to alleviate sexual dysfunction in patients with cancer.^7^ The choice of domains was based on the state of knowledge and experience from clinical practice regarding common problems in young adults following cancer treatment. The following domains were answered by women: Vaginal Lubrication, Vaginal Discomfort, Vulvar Discomfort Clitoral, and Vulvar Discomfort–Labial. The Erectile Function domain was answered by men. Four domains were answered by all participants: Interest in Sexual Activity, Satisfaction with Sex Life, Orgasm–Ability, and Orgasm–Pleasure.

### Participants and procedure

The present study is part of a larger research program investigating concerns related to sexual function and fertility in young adults diagnosed with cancer in Sweden.^6^^,^^7^ The psychometric properties of the Swedish version of the SexFS measure were as a result investigated in a large-scale survey of a clinical population of young adult women and men diagnosed with cancer (breast cancer and testicular cancer, respectively)^11^^,^^12^ and a nonclinical population of young adult women and men from the general population.^13^

Women diagnosed with invasive breast cancer at the age of 18-39 years in Sweden^14^ were identified by use of the Swedish National Quality Register for Breast Cancer, a high-quality register with almost complete nationwide coverage.^15^ Men diagnosed with testicular cancer at the age of 18-39 years in Sweden^11^ were identified by use of the Swedish nationwide high-quality register for testicular cancer, SWENOTECA. This register has a coverage of 98% of all new cases of testicular cancer in Sweden.^16^ For women and men in the clinical samples, information on vital status and contact details were obtained through linkage with the Swedish population register.^11^^,^^12^ Information about the study was sent by postal mail to potential participants together with a survey and a prepaid envelope for survey return. At the time of the study, the participants were 1.5 to 2.5 years postdiagnosis.

Participants in the general population sample were identified using the Swedish population register, which covers all persons registered as residents in Sweden. The Swedish population register provided a random sample of 2000 individuals (1000 women and 1000 men) in the age group 19-40 years. The participants were approached via regular mail including a letter with information about the study, a prepaid envelope for survey return, and the survey with the self-administered measures. More detailed information on data collection and characteristics of participants in the clinical and nonclinical samples is available in previous publications.^11–13^

By responding to the survey, participants gave their consent to participate in the study. Written informed consent was thereby given by all participants in the study. Ethical approval of the study was obtained from the Regional Ethical Review Board in Stockholm (Ethical approval number: 2013/1746-31/4; 2014/2244-32; 2017/916-32; 2016/1848-32).

### Statistical analyses

Respondent sociodemographic characteristics were summarized using descriptive statistics (frequencies, percentages, means, SDs). Descriptive statistics for each of the domains of the Swedish version of the SexFS v2.0 were calculated with means and SDs.

The analyses were performed separately for the 4 respondent groups. All respondents were included in the analysis of item and scale properties of the domain Interest in Sexual Activity, but descriptive statistics and psychometric properties of the other domains were based only on the respondents who had answered that they had engaged in sexual activity in the last 30 days.

#### Data quality

Data quality was assessed by evaluating whether all response alternatives were used for all items, as well as floor and ceiling effects. Ceiling effects were measured by the proportion of participants rating the highest possible score, and floor effects were measured by the proportion of participants rating the lowest possible score. The criteria of low or reasonable floor/ceiling effects was set to <15% for the clinical group,^17^ and <25% for the general population, where we expect a higher proportion reporting no problems. Furthermore, we examined the percentage of missing values for each item across domains. A proportion of >5% missing data for an item was found relevant and applied to indicate a sign of problems with feasibility/acceptability.^18^

#### Construct validity

Construct validity was measured by using corrected item-total (domain) correlation using cutoff scores ≥0.4 to indicate acceptable correlation.^19^ Item means and SDs were examined and expected to be roughly equivalent within a domain to justify the summation of item scores into domain scores.^20^ The extent to which items in a domain measured the same construct (homogeneity of the domain) was further assessed with item-to-own domain correlations. Scaling assumptions were tested by measuring scaling success as evidence that an item correlated more highly with its own domain than the other domains. Scaling success was calculated by dividing the number of item-to–other domain correlations that were stronger than the corrected item total correlation within a domain with the total number of tests (ie, number of items in own domain × number of other domains). A scaling success of 100 means that no item-to–other domain correlations were stronger than the corrected item total correlation.

#### Reliability

Reliability was measured by estimation of internal consistency, using Cronbach’s α coefficient, with α of 0.70-0.90 suggested to reflect adequate internal consistency,^20^ where 0.7 is the standard cutoff for group comparisons and 0.9 is the standard cutoff for individual comparisons.^21^

All analyses were conducted using IBM SPSS Software version 28 (IBM Corp.).

## Results

### Participants

Characteristics of the participants are presented in Table 2. The SexFS measure was answered by 180 women with breast cancer (60% response rate) and by 511 women from the Swedish general population (51% response rate). Of the responders to the question of having had any sexual activity in the past 30 days, 151 (84%) women with cancer and 442 (86%) women in the nonclinical general population sample reported yes (Table 3).

**Table 2:** Characteristics of the participants who answered the Swedish version of the SexFS v2.0 measure.

	**Women**		**Men**	
	**Breast cancer *n* = 180**	**General population, *n* = 511**	**Testicular cancer, *n* = 110**	**General population, *n* = 324**
Age, years				
Mean (SD)	34.6 (4.0)	29.8 (6.1)	32.1 (5.5)	29.3 (6.4)
Range	22-39	19-40	18-42	19-40
Country of birth, *n* (%)				
Sweden	147 (81.7)	437 (85.5)	100 (90.9)	270 (83.3)
Other	33 (18.3)	73 (14.3)	10 (9.1)	54 (16.7)
Do not want to declare		1 (0.2)		
Education, *n* (%)				
Elementary school	4 (2.2)	18 (3.6)	6 (5.5)	12 (3.7)
High school	61 (33.9)	183 (36.1)	45 (40.9)	153 (47.2)
University	110 (61.1)	294 (57.5)	53 (48.2)	150 (46.3)
Other	5 (2.8)	12 (2.3)	4 (3.6)	8 (2.5)
Do not want to declare		1 (0.2)		
Missing		3 (0.6)	2 (1.8)	1 (0.3)
Main occupation, *n* (%)				
Full-time work	88 (48.9)	294 (57.5)	86 (78.2)	228 (70.4)
Part-time work	52 (28.9)	81 (15.9)	7 (6.4)	24 (7.4)
Studying	16 (8.9)	79 (15.5)	8 (7.3)	52 (16.0)
Unemployed	6 (3.3)	14 (2.7)	1 (0.9)	6 (1.9)
Sick leave	14 (7.8)	21 (4.1)	5 (4.5)	6 (1.9)
Other	4 (2.2)	20 (3.9)	3 (2.7)	6 (1.9)
Do not want to declare		1 (0.2)		
Missing		1 (0.2)		2 (0.6)
Partnered, *n* (%)				
Yes	157 (87.2)	408 (79.8)	90 (81.8)	232 (71.6)
Have children, *n* (%)				
Yes	137 (76.1)	244 (47.8)	53 (48.2)	127 (39.2)
Sexual orientation, *n* (%)				
Heterosexual	168 (93.3)	466 (91.2)	104 (94.5)	306 (94.4)
Gay or lesbian	1 (0.6)	7 (1.4)	3 (2.7)	8 (2.5)
Bisexual	4 (2.2)	26 (5.1)	1 (0.9)	7 (2.2)
Other	2 (1.1)	4 (0.8)		3 (0.9)
Do not want to declare	2 (1.1)	5 (1.0)	1 (0.9)	
Missing	3 (1.7)	3 (0.6)	1 (0.9)	

**Table 3:** Descriptive and psychometric statistics for the PROMIS SexFS measure in clinical and nonclinical populations.

**Domain (number of items)**	***n***	**No sexual activity in the past 30 days, *n* (%)** ^**a**^	***n*** ^**b**^	**Mean (SD)**	**Range of item, mean (SD)**	**Floor/ceiling effect, %**	**Reliability, α**	**Item-to own–domain correlation, range**	**Item-to–other domain correlation, range**	**Scaling success, %** ^**c**^
**Women With Breast Cancer**										
Interest (2)	180	NA	179	2.64 (0.99)	2.61-2.68 (0.91-1.24)	8.9/0	0.80	0.90-0.95	−0.49-0.65	100
Lubrication (2)	151	3 (2.0)	148	3.64 (1.28)	3.53-3.74 (1.31-1.43)	7.4/26.4	0.87	0.93-0.94	−0.57-0.54	100
Vaginal Discomfort (4)	151	3-4 (2.0-2.6)	144	1.83 (0.97)	1.50-2.05 (0.94-1.38)	37.5/0.7	0.87	0.48-0.95	−0.60-0.68	96
Satisfaction With Sex Life (2)	151	NA	150	3.05 (1.26)	2.94-3.17 (1.26-1.44)	6.0/15.3	0.84	0.92-0.94	−0.47-0.66	100
Orgasm-Pleasure (1)	151	15 (9.9)	136	3.90 (1.16)	NA	2.9/41.2	NA	NA	−0.29-0.74	NA
Orgasm-Ability (1)	151	2 (1.3)	149	3.60 (1.31)	NA	9.4/34.2	NA	NA	−0.39-0.74	NA
Vulvar Discomfort-Clitoral (1)	151	2 (1.3)	149	1.50 (0.93)	NA	69.8/2.7	NA	NA	−0.43-0.60	NA
Vulvar Discomfort- Labial (1)	151	3 (2.0)	146	1.68 (1.06)	NA	59.6/3.5	NA	NA	−0.54-0.68	NA
**Women in the General Population**										
Interest (2)	510	NA	506	3.21 (1.00)	3.07-3.36 (0.88-1.25)	3.2/2.4	0.83	0.91-0.96	−0.06-0.46	100
Lubrication (2)	442	10 (2.3)	430	4.49 (0.79)	4.47-4.50 (0.86-0.93)	1.4/54.9	0.75	0.89-0.90	−0.32-0.40	100
Vaginal Discomfort (4)	442	6-8 (1.4-1.8)	431	1.46 (0.59)	1.34-1.64 (0.68-0.87)	43.6/0	0.79	0.46-0.89	−0.31-0.39	96
Satisfaction With Sex Life (2)	442	NA	440	3.75 (1.06)	3.68-3.82 (1.06-1.19)	1.8/23.9	0.87	0.93-0.95	−0.20-0.48	100
Orgasm–Pleasure (1)	442	36 (8.2)	404	4.25 (0.86)	NA	0.5/46.8	NA	NA	−0.10-0.76	NA
Orgasm–Ability (1)	442	19 (4.3)	422	3.94 (1.10)	NA	4.7/37.9	NA	NA	−0.08-0.76	NA
Vulvar Discomfort Clitoral (1)	442	7 (1.6)	435	1.19 (0.50)	NA	84.8/0	NA	NA	−0.17-0.37	NA
Vulvar Discomfort– Labial (1)	442	8 (1.6)	433	1.25 (0.61)	NA	80.6/0.7	NA	NA	−0.22-0.40	NA
**Men with Testicular Cancer**										
Interest (2)	110	NA	109	3.69 (0.94)	3.56-3.82 (0.85-1.15)	0.9/9.2	0.83	0.91-0.95	0.39-0.54	100
Erectile Function (3)	106	0-14 (0-13.2)	91	4.56 (0.77)	4.51-4.74 (0.60-1.03)	0.0/63.7	0.79	0.82-0.89	0.20-0.71	100
Satisfaction With Sex Life (2)	106	NA	105	3.56 (1.21)	3.43-3.70 (1.19-1.32)	5.7/18.1	0.92	0.96-0.97	0.37-0.65	100
Orgasm–Pleasure (1)	106	1 (0.9)	104	4.04 (0.87)	NA	0.0/31.7	NA	NA	0.39-0.66	NA
Orgasm–Ability (1)	106	0	105	4.58 (0.81)	NA	1.9/71.4	NA	NA	0.40-0.66	NA
**Men in the General Population**										
Interest (2)	324	NA	323	3.77 (0.81)	3.56-3.97 (0.72-1.07)	1.2/4.0	0.74	0.86-0.94	0.01-0.40	100
Erectile Function (3)	301	5-67 (1.7-22.3)	230	4.77 (0.53)	4.74-4.81 (0.62-0.85)	0.0/78.3	0.53	0.62-0.79	−0.09-0.22	100
Satisfaction With Sex Life (2)	301	NA	301	3.58 (1.19)	3.45-3.72 (1.19-1.34)	3.0/22.9	0.87	0.93-0.94	−0.03-0.52	100
Orgasm–Pleasure (1)	301	5 (1.7)	296	4.08 (0.88)	NA	0.7/35.8	NA	NA	0.17-0.54	NA
Orgasm–Ability (1)	301	6 (2.0)	294	4.64 (0.66)	NA	0.7/71.4	NA	NA	−0.01-0.30	NA

NA, not applicable.

a Depending on the type of question: Haven’t had sex/intercourse/an orgasm/tried to orgasm in the past 30 days.

b Number of respondents that the mean value of the scale and analyses of scale properties were based on.

c Number of item-to–other domain correlations that are stronger than the corrected item-total correlation within a domain/total number of discriminant validity tests (ie, number of items in own domain × number of other domains), expressed as a percentage.

The SexFS measure was answered by 110 men with testicular cancer (50% response rate), and 324 men from the general population sample (34% response rate). Of the responders to the question of having had any sexual activity, 106 (96%) men with cancer and 301 (93%) men in the nonclinical general population sample reported yes (Table 3).

### Data quality

The distribution of answers for each item and respondent group is shown in Figure 1 A-C. Among women, all response alternatives were used for all items, except for the item concerning Vulvar Discomfort Clitoral (“When you have had sexual activity, how much discomfort have you had in your clitoris?”), for which no woman in the general population reported “A lot,” corresponding to a 5 on the scale (Figure 1B). Among men, all response alternatives were used for all items except for the Orgasm–Pleasure item “How satisfying has your orgasm been?”, for which no man with cancer reported “Not at all,” corresponding to a 1 on the scale (Figure 1B), and the item “How often were you able to have an erection during sexual activity?,” for which no man with cancer responded “Almost never/never,” corresponding to a 1 on the scale (Figure 1C).

**Figure 1:**
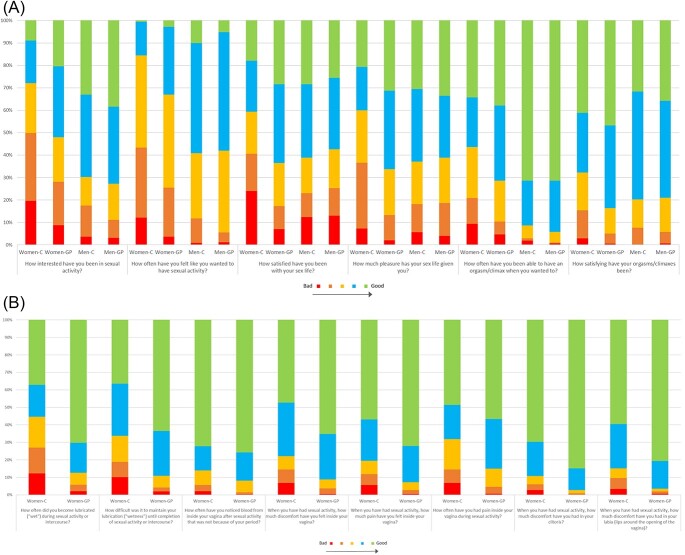
Distribution of answers for the participants on the generic items (A), body part–specific items answered by women (B), and body part–specific items answered by men (C). All items on 5-point Likert scales that denote “bad” to “good” function. Some items have been reversed to facilitate comparisons among items. Women-C, women with breast cancer; Women-GP, women in the general population; Men-C, men with testicular cancer; Men-GP, men in the general population.

**Figure 1:**
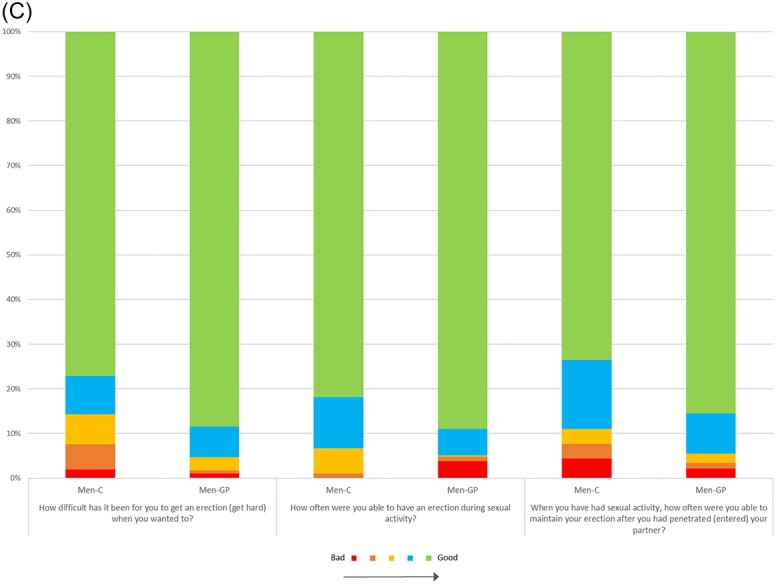
Continued.

Descriptive statistics including floor and ceiling effects are presented in Table 3. Among female respondents in the clinical and nonclinical group, floor effects ranged between 1% and 85%, and ceiling effects ranged between 0% and 55%. Among male participants, floor effects ranged between 0% and 6%, and ceiling effects ranged between 4% and 78%. For both female and male respondents in the clinical and nonclinical group, floor or ceiling effects of >15% and >25%, respectively, were found for all domains except for the Interest in Sexual Activity and Satisfaction With Sex Life for women and men in the general population.

Among women in the clinical and nonclinical group, missing responses ranged from 0 to 3 per item (proportion of missing: 0%-3%) within domains. Prevalence of missing data was also low (<1%) among men in both groups.

### Construct validity

Item means within domains were roughly equivalent, and the SDs were close to one. Among women, the corrected item-total correlation coefficients were between 0.60 and 0.90, thus showing adequate correlation, for all items except for the item concerning bleeding from inside the vagina after sexual activity. The vaginal bleeding item showed a corrected item-total correlation of 0.27 for women with cancer and 0.19 for women in the nonclinical group (Supplementary Table 2). Among men, corrected item-total correlation coefficients exceeded 0.4, except for the nonclinical group where the items in the Erectile Function domain showed corrected item-total correlation coefficients below the cut-off (0.32-0.38; Supplementary Table 2).

For women in the clinical and nonclinical groups, the scaling success rate of domains with more than 1 item was 100 for all domains except Vaginal Discomfort, reaching a 96% scaling success (Table 3). For men, the scaling success was 100% for all domains.

### Internal consistency

For women in both the clinical and nonclinical groups, Cronbach’s α values for all 4 domains were adequate to good (range: 0.75-0.87). Cronbach’s α values were also adequate to good for men (range: 0.74-0.92), except for Erectile Function, for which a lower Cronbach’s α of 0.53 was found among young men in the nonclinical general population group (Table 3). A sensitivity analysis was therefore conducted on the Erectile Function domain, for which the clinical and nonclinical samples were combined. The analysis of the combined sample showed a Cronbach’s α of the Erectile Function domain of 0.65, reflecting a higher internal consistency (though still below 0.70).

## Discussion

In this study, we evaluated the psychometric properties of the translated and culturally adapted Swedish version of the SexFS measurement tool in young adult women and men from a clinical (patients with cancer) and nonclinical population (general population sample). The Swedish SexFS has been found to show face validity in evaluation with individuals from the target groups as well as older adults (16-70 years old),^9^ and the results of the present study demonstrate reasonable evidence that the Swedish version of the SexFS measure is also feasible, valid, and reliable in the assessing of sexual functioning and satisfaction in young adults.

Our version of the SexFS measure with the given response alternatives is satisfactory for use among young adults who understand the Swedish language. According to the results of item frequency distribution, the response alternatives were utilized and the degree of internal missing data was low, findings which indicate that the measure was found feasible and acceptable in both women and men. We identified moderate to significant floor and ceiling effects in most domains and across respondent groups. For example, both for women in the clinical and those in the nonclinical group, the single-item domains Vulvar Discomfort Clitoral and Labial, respectively, resulted in significant floor effects (60% and 85%), which showed that the majority of women in both groups had no discomfort. Similarly, the identified ceiling effects (64% and 78%) of the domain Erectile Function showed that a substantial proportion of men in both the clinical and nonclinical group didn’t experience problems with erectile function. Considerable floor/ceiling effects typically indicate a lower ability of a measure to assess meaningful variation at the extremes,^17^ in this case between respondents who report high sexual functioning or no discomfort. Results from cognitive interviews and item characteristic curves (based on item response theory) of the SexFS measure both suggest that the response alternatives are sufficient.^2^^,^^5^ However, sample(s) of participants of young age, like the those in the present study, would be expected to have floor/ceiling effects, which the measure appears to reflect accurately.

Our results show that item scores were roughly equivalent across the domain and the corrected item-total correlation coefficient exceeded 0.4 for most items, thus confirming the construct validity of the Swedish version of the SexFS measure.^19^ This finding supports the notion that domain scores can be computed by combining the responses for the items belonging to each domain. However, an item of the Swedish version that did not perform well was the item concerning vaginal bleeding, in the Vaginal Discomfort domain. In both the clinical and nonclinical group, the corrected item-total coefficient of this item indicated inadequate correlation to the scale. Although a scaling success rate of 96% is good, the vaginal bleeding item with its lower corrected item-total coefficients explains why the Vaginal Discomfort domain did not reach 100% scaling success. Vaginal bleeding after sexual activity might be a more relevant issue for older than younger women, as reflected by the scores of the young adult women participants. The SexFS measure is customizable in that items and domains can be selected by the researcher based on the purpose of the study. The items of the Swedish version of the SexFS were retrieved from a preliminary version of the SexFS 2.0 measure. Our results show that the inclusion of the specific item of bleeding from inside the vagina after sexual activity is problematic, which leads to our recommendation not to include this item. Indeed, as supported by our results, this item was dropped in the final version of the SexFS 2.0 measure.^5^

In our study, reliability, as measured by Cronbach’s α, was above the acceptable range in all domains for women in both respondent groups and for men in the clinical groups. Psychometric evidence for the reliability of the Interest in Sex Life and Satisfaction With Sex Life domains was also demonstrated among men in the nonclinical group. This finding is consistent with previous studies conducted in adult cancer patient populations in the United States.^4^^,^^22^ In line with previous studies on men with cancer,^22^ the results provide support for use of the SexFS measure to assess erectile function in our sample of men with testicular cancer. However, in the nonclinical general population group of men there was little variation in item scores and significant ceiling effects, reflecting high functioning in this young adult population. When there is little variance in the item responses, there is less covariance between items, which translates into lower correlations, as seen the low item-total correlation coefficients in this group. Since Cronbach’s α is a function of the average inter-item correlation, Cronbach’s α was also lower. Clinically, it is reassuring that very few young men in the general population reported problems with erectile function.

A strength of the present study is the nationwide population-based sample of young adult patients with breast or testicular cancer, identified from national quality registers, which minimized selection bias related to problems with sexual functioning. The inclusion of women and men from the general population is another strength that allowed for insights into the psychometric properties of the measure in both clinical and nonclinical populations. There are also some limitations to consider. One limitation relates to the response rate in each respondent group, which introduces a risk of bias in estimates that should be taken into consideration when interpreting the results. As expected based on previous studies on the general population, men in the general population had a lower response rate (34%) compared to the other respondent groups. It is possible that the topic of sexual functioning was sensitive for some young men in the general population who had sexual difficulties. Also, this study was limited to young adults (aged 19-40 years). Although it cannot be assumed that these results can be generalized to the adult population, the Swedish version of the measure has previously shown face validity when tested on adults older than 40 years.^9^ Furthermore, additional studies are needed to understand how the Swedish version of the PROMIS SexFS works in other targeted populations, for instance other clinical groups such as adults with diabetes, heart disease, or mental illness. Finally, because the psychometric evaluation was performed as part of a larger research program on fertility and sexuality following cancer, no test–retest data were assessed. To further strengthen the evidence of reliability, future studies should examine the test–retest consistency over time of the Swedish version of the SexFS measure.

## Conclusions

The results of this study suggest that the Swedish version of the SexFS measure is a valid, reliable and flexible tool for researchers and clinicians in Sweden to measure self-reported sexual function and satisfaction in men and women and specifically to identify individuals who experience sexual problems and who might benefit from targeted interventions.

## Supplementary Material

CLEAN_Supplemental_table_1_qfac006

Supplemental_table_2_qfac006
