# Supplementary material for: Psychometric evaluation of the Swedish version of the PROMIS Sexual Function and Satisfaction Measures in clinical and nonclinical young adult populations
Source: Sex Med. 2023 Jan 12;11(1):qfac006. doi: 10.1093/sexmed/qfac006 (PMC10065179; doi:10.1093/sexmed/qfac006)
Supplement: CLEAN_Supplemental_table_1_qfac006 [file clean_supplemental_table_1_qfac006.docx]

**Supplemental material**

| **Table S1.** Overview of domains and items of the Swedish version of the Patient-Reported Outcomes Measurement Information System (PROMIS)® Sexual Function and Satisfaction measure (SexFS) version 2.0.  Please note that use any items of the SexFS requires permission of and registration at [HealthMeasures - PROMIS Health Organization](https://www.promishealth.org/healthmeasures/)**.** | | | |
| --- | --- | --- | --- |
| **Domains** | **Items (No)** | **Original version** | **Swedish version** |
| Sexual activity screener items | 1 | **In the past 30 days, did you have any type of sexual activity?** (Examples of sexual activity are masturbation, oral sex, and sexual intercourse)     - No - Yes | **De senaste 30 dagarna, har du haft någon typ av sexuell aktivitet?** (Exempel på sexuell aktivitet är onani, oralsex och samlag)   - Nej - Ja |
| Interest in sexual activity | 2 | **How interested have you been in sexual activity?**  1) Not at all  2) A little bit  3) Somewhat  4) Quite a bit  5) Very | **Hur intresserad har du varit av sexuell aktivitet?**  1) Inte alls  2) Lite grann  3) Till viss del  4) Ganska  5) Väldigt |
|  |  | **How often have you felt like you wanted to have sexual activity?**  1) Never  2) Rarely  3) Sometimes  4) Often  5) Always | **Hur ofta har du känt att du velat ha sexuell aktivitet?**  1) Aldrig  2) Sällan  3) Ibland  4) Ofta  5) Alltid |
| Satisfaction with sex life | 2 | **How satisfied have you been with your sex life?**  1) Not at all  2) A little bit  3) Somewhat  4) Quite a bit  5) Very | **Hur nöjd har du varit med ditt sexliv?**  1) Inte alls  2) Lite grann  3) Till viss del  4) Ganska  5) Väldigt |
|  |  | **How much pleasure has your sex life given you?**  1) None  2) A little bit  3) Some  4) Quite a bit  5) A lot | **Hur mycket njutning har ditt sexliv gett dig?**  1) Ingen  2) Lite grann  3) En del  4) En hel del  5) Mycket |
| Orgasm - Ability | 1 | **How often have you been able to have an orgasm/climax when you wanted to?**  0) Have not tried to have an orgasm/climax in the past 30 days  1) Never  2) Rarely  3) Sometimes  4) Often  5) Always | **Hur ofta har du kunnat få orgasm när du velat det?**  0) Har inte försökt få orgasm de senaste 30 dagarna  1) Aldrig  2) Sällan  3) Ibland  4) Ofta  5) Alltid |
| Orgasm - Pleasure | 1 | **How satisfying have your orgasms or climaxes been?**  0) Have not had an orgasm/climax in the past 30 days  1) Not at all  2) A little bit  3) Somewhat  4) Quite a bit  5) Very | **Hur tillfredsställande har dina orgasmer varit?**  0) Har inte haft orgasm de senaste 30 dagarna  1) Inte alls  2) Lite grann  3) Till viss del  4) Ganska  5) Väldigt |

| Vaginal Lubrication (women) | 2 | **How often did you become lubricated ("wet") during sexual activity or intercourse?**  0) No sexual activity in the past 30 days  1) Almost never or never  2) A few times (less than half the time)  3) Sometimes (about half of the time)  4) Most times (more than half the time)  5) Almost always or always | **Hur ofta blev du fuktig ("våt") vid sexuell aktivitet eller samlag?**  0) Ingen sexuell aktivitet de senaste 30 dagarna  1) Nästan aldrig eller aldrig  2) Vid några tillfällen (mindre än hälften av tiden)  3) Ibland (ungefär hälften av tiden)  4) Oftast (mer än hälften av tiden)  5) Nästan alltid/alltid |
| --- | --- | --- | --- |
|  |  | **How difficult was it to maintain your lubrication ("wetness") until completion of sexual activity or intercourse?**   1. No sexual activity in the past 30 days   1) Extremely difficult or impossible  2) Very difficult  3) Difficult  4) Slightly difficult  5) Not difficult | **Hur svårt var det att bibehålla din fuktighet (förbli "våt") till dess att sexuell aktivitet eller samlag fullbordats?**   1. Ingen sexuell aktivitet de senaste 30 dagarna   1) Extremt svårt eller omöjligt  2) Väldigt svårt  3) Svårt  4) Lite svårt  5) Inte svårt |
| Vaginal Discomfort (women) | 4 | **How often have you noticed blood from inside your vagina after sexual activity that was not because of your period?**   1. Have not had sexual activity in the past 30 days   1) Never  2) Rarely  3) Sometimes  4) Often  5) Always | **Hur ofta har du lagt märke till blod inifrån slidan efter sexuell aktivitet som inte berodde på mens?**   1. Har inte haft sexuell aktivitet de senaste 30 dagarna   1) Aldrig  2) Sällan  3) Ibland  4) Ofta  5) Alltid |
|  |  | **When you have had sexual activity, how much discomfort have you felt inside your vagina?**   1. Have not had sexual activity in the past 30 days   1) None  2) A little bit  3) Some  4) Quite a bit  5) A lot | **När du har haft sexuell aktivitet, hur mycket obehag har du känt inne i din slida?**   1. Har inte haft sexuell aktivitet de senaste 30 dagarna   1) Inget  2) Lite grann  3) En del  4) En hel del  5) Mycket |

|  |  | **When you have had sexual activity, how much pain have you felt inside your vagina?**  0) Have not had sexual activity in the past 30 days  1) None  2) A little bit  3) Some  4) Quite a bit  5) A lot | **När du har haft sexuell aktivitet, hur mycket smärta har du känt inne i slidan?**   1. Har inte haft sexuell aktivitet de senaste 30 dagarna   1) Ingen  2) Lite grann  3) En del  4) En hel del  5) Mycket |
| --- | --- | --- | --- |
|  |  | **How often have you had pain inside your vagina during sexual activity?**   1. Have not had sexual activity in the past 30 days   1) Never  2) Rarely  3) Sometimes  4) Often  5) Always | **Hur ofta har du haft smärta inne i slidan vid sexuell aktivitet?**   1. Har inte haft sexuell aktivitet de senaste 30 dagarna   1) Aldrig  2) Sällan  3) Ibland  4) Ofta  5) Alltid |
| Vulvar Discomfort Clitoral (women) | 1 | **When you have had sexual activity, how much discomfort have you had in your clitoris (clit)?**   1. No sexual activity in the past 30 days   1) None  2) A little bit  3) Some  4) Quite a bit  5) A lot | **När du har haft sexuell aktivitet, hur mycket obehag har du haft i din klitoris?**   1. Har inte haft sexuell aktivitet de senaste 30 dagarna   1) Inget  2) Lite grann  3) En del  4) En hel del  5) Mycket |
| Vulvar Discomfort Labial (women) | 1 | **When you have had sexual activity, how much discomfort have you had in your labia (lips around the opening of the vagina)?**   1. Have not had sexual activity in the past 30 days   1) None  2) A little bit  3) Some  4) Quite a bit  5) A lot | **När du har haft sexuell aktivitet, hur mycket obehag har du haft i dina blygdläppar (läpparna runt slidöppningen)?**   1. Har inte haft sexuell aktivitet de senaste 30 dagarna   1) Inget  2) Lite grann  3) En del  4) En hel del  5) Mycket |

| Erectile Function (men) | 3 | **How difficult has it been for you to get an erection or (get hard) when you wanted to?** If you use any aids to help you get an erection (e.g., pills, injections, or a penis pump) please answer this question thinking about the times that you used these aids.  0) Have not tried to get an erection (get hard) in the past 30 days  1) Very  2) Quite  3) Somewhat  4) A little  5) Not at all | **Hur svårt har det varit för dig att få erektion (stånd) när du har velat?** Om du använder några hjälpmedel för att få erektion (t.ex. tabletter, injektioner eller penispump), så utgå från de tillfällen när du använt dessa hjälpmedel, när du besvarar frågan.   1. Har inte försökt få erektion (stånd) de senaste 30 dagarna   1) Väldigt  2) Ganska  3) Till viss del  4) Lite grann  5) Inte alls |
| --- | --- | --- | --- |
|  |  | **How often were you able to get an erection during sexual activity?**  0) No sexual activity in the past 30 days  1) Almost never/never  2) A few times (less than half of the times)  3) Sometimes (about half of the times)  4) Most times (more than half the times)  5) Almost always/always | **Hur ofta hade du förmåga att få erektion vid sexuell aktivitet?**  0) Har inte haft någon sexuell aktivitet de senaste 30 dagarna  1) Nästan aldrig/aldrig)  2) Vid några tillfällen (mindre än hälften av gångerna  3) Ibland (ungefär hälften av gångerna)  4) Oftast (mer än hälften av gångerna)  5) Nästan alltid/alltid |
|  |  | **During sexual intercourse how often were you able to maintain your erection after you had penetrated (entered) your partner?**  0) Did not attempt intercourse  1) Almost never/never  2) A few times (less than half of the times)  3) Sometimes (about half of the times)  4) Most times (more than half the times)  5) Almost always/always | **Under samlag, hur ofta kunde du behålla din erektion efter att du hade penetrerat (trängt in i) din partner?**  0) Försökte inte ha samlag  1) Nästan aldrig/aldrig  2) Vid några tillfällen (mindre än hälften av gångerna)  3) Ibland (ungefär hälften av gångerna)  4) Oftast (mer än hälften av gångerna)  5) Nästan alltid/alltid |
