# Supplementary material for: Psychometric evaluation of the Swedish version of the PROMIS Sexual Function and Satisfaction Measures in clinical and nonclinical young adult populations
Source: Sex Med. 2023 Jan 12;11(1):qfac006. doi: 10.1093/sexmed/qfac006 (PMC10065179; doi:10.1093/sexmed/qfac006)
Supplement: Supplemental_table_2_qfac006 [file supplemental_table_2_qfac006.docx]

| **Table** **S2.** Corrected item-total (scale) correlation of SexFS domains with more than one item | | | | |
| --- | --- | --- | --- | --- |
| **Domain and item** | **Women with breast cancer** | **Women in the general population** | **Men with testicular cancer** | **Men in the general population** |
| **Interest in Sexual Activity** |  |  |  |  |
| - How interested have you been in sexual activity? | 0.70 | 0.76 | 0.74 | 0.64 |
| - How often were you interested to have a sexual activity? | 0.70 | 0.76 | 0.74 | 0.64 |
| **Satisfaction with Sex Life** |  |  |  |  |
| - How satisfied have you been with your sex life? | 0.72 | 0.77 | 0.85 | 0.77 |
| - How much pleasure has your sex life given you? | 0.72 | 0.77 | 0.85 | 0.77 |
| **Vaginal Lubrication (women)** |  |  |  |  |
| - How often did you your vagina get moist ('wet') during sexual activity or intercourse? | 0.78 | 0.60 | N/A | N/A |
| - How hard was it to keep the vagina moist ('wet') during sexual activity or intercourse? | 0.78 | 0.60 | N/A | N/A |
| **Vaginal Discomfort (women)** |  |  |  |  |
| - How often have you noticed vaginal bleeding after sexual activity that was not part of a regular period? | **0.27** | **0.19** | N/A | N/A |
| - During sexual activity, how much vaginal discomfort have you felt? | 0.88 | 0.71 | N/A | N/A |
| - During sexual activity, how much vaginal pain have you felt? | 0.90 | 0.78 | N/A | N/A |
| - How often have you experienced vaginal pain during sexual activity? | 0.88 | 0.76 | N/A | N/A |
| **Erectile function (men)** |  |  |  |  |
| - How difficult has it been for you to get an erection or get hard when you wanted to? | N/A | N/A | 0.60 | **0.32** |
| - How often were you able to get an erection during sexual activity? | N/A | N/A | 0.69 | **0.38** |
| - During sexual intercourse how often were you able to maintain your erection after you had penetrated (entered) your partner? | N/A | N/A | 0.70 | **0.37** |
| **Note**. Bold values denote coefficients below the ≥0.4 cut-off (values below indicate inadequate correlation) | | | | |
